# Shared Decision Making Between Older Adult Home Health Patients and Their Caregivers: A Dyadic Coping Perspective

**DOI:** 10.1093/geroni/igaa057.1156

**Published:** 2020-12-16

**Authors:** Djin Tay, Lee Ellington, Gail Towsley, Katherine Supiano, Cynthia Berg

**Affiliations:** University of Utah, Salt Lake City, Utah, United States

## Abstract

Older adult Home health (HH) patients comprise a medically frail population with increased inpatient and emergency department utilization. Despite the need for advance care planning among this population, rates are suboptimal. Patients rely increasingly on caregivers to advocate and coordinate their care particularly at the end of life; however surrogate decision makers are often underprepared for their roles in end-of-life decision making. This study examined shared decision making processes among older adult HH patients and caregivers during a shared decision making intervention guided by the Developmental-Contextual Model of dyadic coping (DCM). Purposive recruitment of N=18 HH patient-caregiver dyads was conducted. Patients were 55 years and above and participated with a family or non-family caregiver they nominated to the study. A 10-41 minute long video-recorded advance care planning intervention was conducted in patients’ homes and analyzed for non-verbal and verbal interactions using Noldus Observer XT 14.0. Theoretically-derived codes were applied deductively in a content analysis to examine dyadic processes associated with interactions suggesting agreement (convergent interactions) and disagreement (divergent interactions). Convergent interactions demonstrated greater alignment in illness representations and shared appraisals, and processes involving support, negotiation, and confirmation of preferences were noted. Convergent interactions also facilitated joint planning for future decisions. Disagreement on illness representations and/or shared appraisals, and overriding another’s preference was observed with divergent interactions. This study builds the groundwork for intervention refinement to promote constructive decision making and address non-constructive decision making among patient and caregivers for advance care planning.

